# Fractal Silver Dendrites as 3D SERS Platform for Highly Sensitive Detection of Biomolecules in Hydration Conditions

**DOI:** 10.3390/nano9111630

**Published:** 2019-11-16

**Authors:** Maria José Lo Faro, Cristiano D’Andrea, Antonio Alessio Leonardi, Dario Morganti, Alessia Irrera, Barbara Fazio

**Affiliations:** 1 Dipartimento di Fisica e Astronomia, Università di Catania, via S. Sofia 64, 95123 Catania, Italy; mariajose.lofaro@dfa.unict.it (M.J.L.F.); antonio.leonardi@ct.infn.it (A.A.L.); dario.morganti@ct.infn.it (D.M.); 2 CNR - IPCF, Istituto per I Processi Chimico-Fisici, viale F. Stagno d’Alcontres 37, 98158 Messina, Italy; 3 CNR - MATIS IMM, Istituto per la Microelettronica e Microsistemi, via S. Sofia 64, 95123 Catania, Italy; 4 CNR - IFAC, Istituto di Fisica Applicata “Nello Carrara”, Via Madonna del Piano, 10, I-50019 Sesto Fiorentino, Italy; c.dandrea@ifac.cnr.it

**Keywords:** Ag dendrites, fractal, SERS, hydrated protein, lysozyme

## Abstract

In this paper, we report on the realization of a highly sensitive and low cost 3D surface-enhanced Raman scattering (SERS) platform. The structural features of the Ag dendrite network that characterize the SERS material were exploited, attesting a remarked self-similarity and scale invariance over a broad range of length scales that are typical of fractal systems. Additional structural and optical investigations confirmed the purity of the metal network, which was characterized by low oxygen contamination and by broad optical resonances introduced by the fractal behavior. The SERS performances of the 3D fractal Ag dendrites were tested for the detection of lysozyme as probe molecule, attesting an enhancement factor of ~2.4 × 10^6^. Experimental results assessed the dendrite material as a suitable SERS detection platform for biomolecules investigations in hydration conditions.

## 1. Introduction

In the last few decades, the task of analytic detection has been extensively studied for different application fields, especially for biomolecules and specific markers, towards the analysis of dangerous analytes [1]. Most commercial sensors rely on the recognition of the target through its fluorescent labeling by using dyes [2] or quantum dots [3]. Despite their diffusion due to the high sensitivity, the use of fluorescent tags introduces complex issues related to their stability due to aggregation, chemical treatment and photobleaching, resulting in a poor reliability of the sensors [4]. Furthermore, the use of labeling in these techniques can introduce a change in the target molecule activity, which can affect their right detection.

For these reasons, an increasing interest has been focused on the realization of sensors with improved characteristics, including the direct recognition of the target species without the need of their marking (label-free). In this context, new contactless optical methodologies are making inroads [5,6,7], requiring the discovery and invention in different fields, from optics to electronics and from chemistry to material science. In particular, nanotechnology provides a valid tool for these optical approaches [8,9]. A suitable example is the setup of new photoluminescent, silicon nanowires sensors [10,11] based on the quenching of optical signals, which allows for the detection of biomolecules with sensitivity down to the femtomolar range. Moreover, the use of metal nanoparticles and the exploitation of the giant electromagnetic field enhancement (10^4^–10^8^), due to the excitation of localized surface plasmon resonances (LSPRs) on their surfaces, allow for the realization of surface enhanced Raman scattering (SERS) sensors [12,13,14,15,16]. Among the optical sensor strategies for label free detection, those based on SERS take this advantage to directly access the target molecules activities, the Raman spectrum representing a molecular fingerprint [17,18,19,20]. This occurrence turns out to be of great relevance in biomedical applications. Hence, the SERS approach has arisen as a rapid sensing platform that is easy to implement and rich in precious spectroscopic information, thus eluding the issue of low Raman scattering cross sections of biomolecules, especially when excited in the visible range [21].

Moreover, in the special conditions of optical coupling between metal nanostructures, the electromagnetic field enhancement can reach huge values (up to 10^12^–10^14^) in spatial regions lying at the interstices between nanoparticles (called hot spots), increasing the sensitivity up to single molecule detection [22,23]. Several methodologies have been adopted with the primary goal of achieving efficient SERS platforms. Among them, it is worth mentioning the realization of SERS substrates that operate in three dimensions (3D), as obtained by decorating high density arrays of silicon nanowires with metal nanoparticles [24,25,26], which allow for the achievement a huge density of hot spots under the laser beam. The major advantage of 3D SERS platforms is related to a significant increment of their surface-to-volume ratio, leading, in the end, to an increased enhancement of factors of at least one order of magnitude compared to lower dimensional SERS substrates [24,27,28]. Among the possible 3D geometries, the characteristics of the self-similarities and scale invariance typical of fractals open the perspective of interesting optical properties, such as broad plasmon resonances arising from the randomness of the structures [29,30]. Additionally, in fractals, the spatial localization of the running waves, not the eigenfunctions of the dilation symmetry operator, can easily promote inhomogeneous electromagnetic field localization, thus leading to the formation of hot spots regions. Indeed, most of the reported SERS enhancement factors (EFs) for fractal common designs have been observed within the range of 10^4^–10^8^, generally outperforming non-fractal geometries performances [27,31,32].

As an example, SERS active platforms based on 3D-fractal Au and Ag octahedral microstructures obtained by lithography and the anisotropic chemical etching of silicon have demonstrated an EF of about 3.51 × 10^5^ by using Rhodamine 6G (R6G, 1 μM) as a probe, one order of magnitude higher than what has been observed for the deposition of the same Au/Ag microstructures on a flat glass surface [28]. Similarly, Docoslis et al. reported on the SERS performances of synthetic Ag dendrites (38% surface coverage) produced an EF of 4 × 10^5^ in situ through an electric field-guided assembly process of colloidal Ag nanoparticles, as estimated by using R6G [33]. Moreover, Cheng et al. demonstrated that Ag dendrite fractals obtained by electrochemical deposition exhibit multiple plasmon resonances, which lead to an enhanced second harmonic generation (SHG) and to improved SERS sensitivity for the detection of 1,4-benzenedithiol (1,4-BDT), used as probe molecule, down to a molar concentration of 10^−14^ M—approximately 77 times larger than that of the non-fractal nanoplates [34]. These examples are representative of the role of Ag fractal aggregates for SERS performances.

On the other hand, the synthesis of Ag fractal dendrites has been well reported in the literature since late 1990s, mainly employing different major approaches based on electrochemical or chemical synthesis by using template or templateless methods for the controlled growth of Ag dendrites [35]. As far as their electrochemical synthesis is concerned, Ag dendrites can be obtained in an electrochemical cell by controlling the Ag reduction onto the substrate electrode. Ag dendrites shape and size are determined by the applied bias, pH, reaction time, and chosen solution [36]. The trench elongation (below hundreds of nm) and branches orientation can be controlled during the growth and can occur from tens of minutes up to a few hours [37]. Nonetheless, this technique presents several drawbacks related to the conductivity of the chosen substrate, requiring costly Pt rods or tubes as counter electrodes. Another limit is the usual growth of asymmetric dendrites subjected to branch breaking and morphological instability after the bias removal due to potentiostatic transitions [38]. Another approach to obtain controlled Ag dendrites in shape and size is through the use of template methods involving Ag salt reduction in the presence of pre-patterned surfaces, such as polymeric templates and porous Raney nickel substrates [39]. By controlling the size and shape of the dendrites, it is possible to tailor their plasmon resonances within specific ranges. However, these template approaches are often diffusion limited, requiring longer synthesis times (from several hours to days) and the use of additional energy to fasten the process, such as voltage [36], microwaves [40], photoactivation [41], ultrasound [37], or catalysts [42]. Moreover, the template removal often required for applications implies the use of aggressive solvents, such as HF or HCl, that can eventually damage the dendrites or introduce contaminations from the template. On the other hand, templateless methodologies are often obtained by means of the random deposition of Ag ions from salt reduction performed in presence of additives, like citrates, sodium borohydride [43], p-aminoazobenzene [44] or Zn microparticles [45], to improve the long reaction time (from hours to days) due to the weak reducing agent. This templateless approach also allows for the control of the dimension and shape of the dendrites by using specific capping agents, like polyvinylpyrrolidone (PVP), during the growth [46]. In particular, the use of capping agents is undesired for SERS applications because it limits the effectiveness of near field enhancements. Thus, in this paper, we present the chemical synthesis of Ag dendrites without capping agents or templates for the realization of the fractal repetition of random structures, opening the perspective of hot spot-recursive formation on a broad wavelength range. Additionally, this approach allows for the synthesis of Ag dendrites at a lower cost by using industrially implementable procedures [47].

This plasmonic material arises as a reaction product during the synthesis procedure of silicon nanowires by means of the metal assisted chemical etching (MACE) technique, and this material is usually removed at the end of process [48,49]. The aim of this paper is the realization of a SERS platform that operates in 3D, based on the as-described prepared fractal silver dendrites. Here, the Ag dendrites are shown to allow for the SERS detection of lysozyme with an enhancement factor (EF) of up to ~10^6^. Moreover, we clearly reveal a SERS signal that unveils the protein in the hydration condition. Recently, a strategy for in situ hot spot formation for the SERS detection of biomolecules in a liquid environment as natural habitat has been realized through use of optical forces [50,51]. As such, the fractal silver dendrites proposed here can be added to the list of SERS platforms able to detect biomolecules without affecting their functionality.

## 2. Materials and Methods

### 2.1. Chemicals and Materials

Hydrofluoric acid (HF) 40% was acquired by Sigma-Aldrich (Saint Louis, MO, USA), while the used silver nitrate (AgNO_3_) 0.05 N was from Scharlau (Barcelona, Spain). Deionized water (18 MΩ·cm) from the Q-Millipore (Darmstadt, Germany) instrument was used for all the prepared aqueous solutions. Commercial 4″ silicon wafers, (100) orientated and n-type doped (1–5 Ω·cm) with 500 μm of thickness, were purchased from Siegert Wafer (Aachen, Germany) and used for this work. Lyophilized lysozyme and Na_2_HPO_4_ and NaH_2_PO_4_ salts for phosphate buffer saline (PBS) were acquired from Sigma-Aldrich [50].

### 2.2. Ag Dendrites Structural and Optical Characterizations

Silver dendrites were obtain by a chemical bath in a AgNO_3_/HF (2:1 *v*/*v*) aqueous solution, and their morphology was later investigated with a ZEISS (Germany) Supra 25 Scanning Electron Microscope [52,53]. The filling factors, the self-similarity and scale invariance of the fractal Ag dendrites were attested by ImageJ software analyses of the SEM microscopies acquired at different magnifications [54]. The compositional characterization of the Ag dendrite platforms were obtained by energy dispersive X-ray spectroscopy (EDX) through the elicited SEM microscope equipped with an EDAX (Mahwah, NJ, USA) detector. The optical resonances of the Ag dendrites were tested by a UV-VIS Perlkin-Elmer (Waltham, MA, USA) spectrometer used in a reflectance configuration equipped with an integrating sphere.

### 2.3. Raman and SERS Analyses

Several solutions with different molarity concentrations of the lysozyme (Lyz) protein were prepared by dissolving a lyophilized powder of lysozyme in a 0.2 M PBS buffer solution (pH 7.2).

The Raman and SERS sensing performances of the Ag dendrite platforms were tested with a Horiba Jobin-Yvon (Kisshoin, Japan) microRaman spectrometer (HR800) equipped with a Peltier-cooled Charge-Coupled Device (CCD) detector (Synapse, Horiba (Kyoto, Japan)). The focused collection of backscattered light was performed by using a 100× (0.9 NA) objective mounted on a BX41 Olympus (Tokyo, Japan) microscope, with an incoming excitation wavelength of 633 nm from a He–Ne laser. Raman investigations were directly performed in 80 µL of a liquid solution of lysozyme in PBS at 10^−2^ M, held in a glass microcell (Marienfled GmbH), and covered with a microscope glass coverslip (Forlab). SERS spectra were collected on Ag dendrite substrates where a micro-droplet of protein solution at concentrations up to 10^−5^ M was pipetted and then left to dry in air overnight.

For all the spectra, we used the same 100× microscope objective with an integration time of 30 s, while the power intensity used was of 45 μW for SERS and 4.5 mW for Raman measurements.

## 3. Results and Discussions

### 3.1. Ag Dendrite Synthesis and Characterization

**Figure 1:**
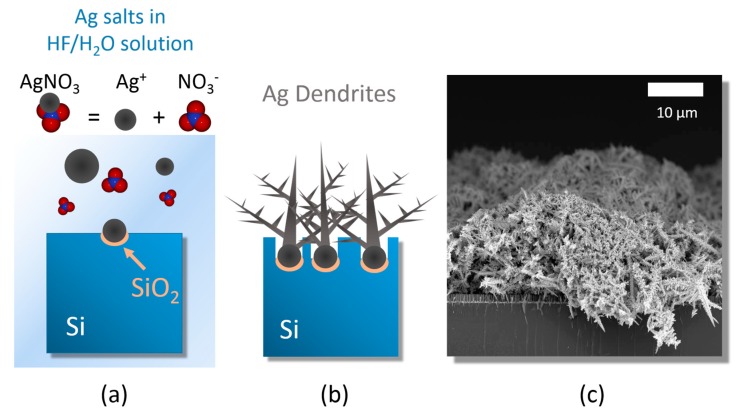
Ag dendrite synthesis. Schematic depicting the growth of Ag dendrites from (**a**) the dissolution of AgNO_3_ salts in a HF/H_2_O solvent, leading to the formation of Ag nanoparticles that subsequently precipitate onto the Si bulk. (**b**) Ag dendrites were developed by further incorporating the Ag^+^ ions onto the initial seeds. (**c**) Cross-section SEM microscopy of the produced carpet of Ag dendrites onto the Si bulk.

The 3D platform of fractal silver dendrites was obtained through the precipitation and subsequent agglomeration of silver salts in a HF watery solution, as sketched in Figure 1.

The silicon wafers were first cleaned from organic contaminants with a 2 min of UV ozone treatments, and then they were etched in a 5% HF aqueous solution for the removal of the native oxide present onto the surface and at the end immersed in a solution containing 0.02 M of silver nitrate (AgNO_3_) as metal precursor and 5 M of HF diluted in deionized water.

The dendrite growth can be considered as a three steps process based on:

(i) silver salt dissolution by Ag reduction;
(ii) random Ag seed aggregation and deposition;
(iii) stem growth onto the Ag nucleation sites [45].

When above the saturation conditions, the silver salts dissociate into individual anions to form a precipitation of silver nanoparticles that are randomly distributed onto the silicon surface (Figure 1a). The additional content of silver nucleates onto the nanoparticles surfaces leads to the formation of dendritic structures to reach the equilibrium condition (Figure 1b) [34,35]. Moreover, underneath the network of silver dendrites, the chemical etching of the silicon substrate does occur at the Ag nanoparticles/Si interface due to their electronegativity difference. As a consequence of the Si etching, the formation of nanowires underneath the Ag network is also achieved during this process [54]. All the adopted etching processes were performed at room temperature for easy integration with Si standard technology.

Figure 1c shows a SEM cross-section of the network of Ag dendrites with an average thickness of 15 ± 5 μm, thus enveloping the array of Si NWs after 30 min of etching. The SEM cross section attests that a very dense carpet of Ag dendrites was obtained by this approach, resulting in a promising 3D disordered porous network of spiked metal nanostructures of great interest for SERS sensing. After their preparation, Ag dendrites were kept in a dry environment under a low vacuum condition of 10^−2^ mbar to prevent detrimental surface oxidation.

### 3.2. Structural and Optical Characterizations

**Figure 2:**
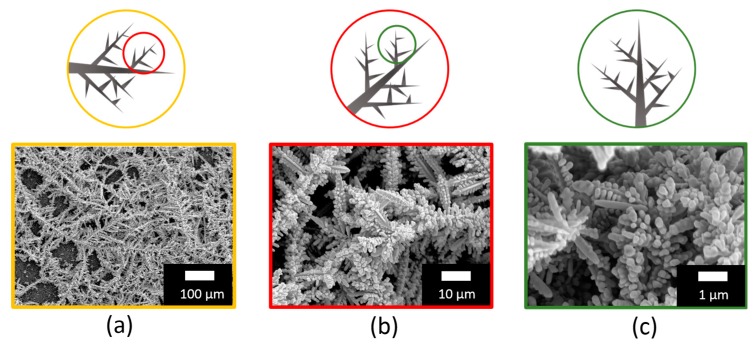
Fractal Ag dendrites: Schematic and plan view scanning electron microscopies of Ag dendrites acquired at three different magnification scales of (**a**–**c**) in order to test the scale invariance of the fractal.

Upon their realization, this complex network of Ag dendrites was characterized through plan view SEM analyses captured at different magnifications of 0.5 kX, 5 kX, and 50 kX, as shown in Figure 2a–c. Indeed, from a first comparison of the three microscopies, it is possible to observe that Figure 2a is characterized by long main trunks with average diameters varying from about 1 to 3 μm that extend from 200 to 400 μm, elongating into smaller secondary or multilevel branches with lengths of about 50 μm, spaced of about 5–15 μm apart. Figure 2b,c also shows the repetition of highly-branched dendrites like those in Figure 2a, but they reported at a higher magnification scale here. This self-similar behavior that was attested over a broad length scale allowed for the investigation of the scale invariance of the system, a typical fingerprint of fractal structures as depicted by the sketch above each figure. Such fractal 3D substrates are promising for SERS investigation due to their increased surface-to-volume ratio and the enhancement of the Raman fingerprint of the target biomolecule due to hot spots formation in the nanogaps [34]. In fact, the Ag dendrites hierarchical structure could be used for efficient light trapping into micro and nanocavities with dimensions over a broad range, thus increasing the SERS detection performance and extending it for a large range of excitation wavelengths.

**Figure 3:**
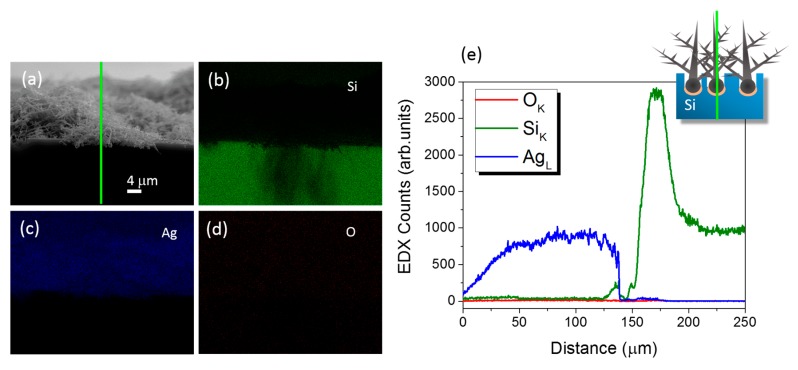
Compositional characterization. Energy dispersive X-ray spectroscopy (EDX) compositional maps of the (**b**) Si (in green), (**c**) Ag (in blue) and (**d**) O (in red) X-ray signals in comparison to (**a**) the SEM cross-section of stored Ag dendrites. (**e**) The EDX elemental profiles acquired for Si, Ag and O X-ray emission lines along the green line reported in (**a**) are also shown.

A critical aspect of Ag dendrites for SERS applications is the presence of surface contaminants due to their storage. Therefore, we performed EDX elemental maps and profile measurements of the stored Ag fractal samples reported in Figure 3 in order to evaluate the metal structures quality and compositions. The Si, Ag and O X-ray emission maps are displayed in green, blue and red in Figure 3b–d, respectively. From their comparison with the SEM cross-section shown in Figure 3a, it can be clearly attested that Ag dendrites were grown onto the Si substrate with good quality, and a neglectable amount of oxygen is shown, both from the Ag dendrites and the Si bulk.

To further investigate the Ag oxidation, EDX profilometry was performed along the vertical section of Ag dendrites onto the Si bulk, as sketched by the green line in Figure 3a. Indeed, the green, blue and red spectra in Figure 3e correspond to the intensity counts from the Si Kα, Ag Lα and O Kα X-ray emission lines, respectively, confirming that the presence of oxygen contamination was negligible on the as-prepared and stored samples.

We further investigated the presence of contaminations and induced oxidation in Ag dendrites as a function of time by means of UV-ozone treatments, as reported in the Supplementary Information (see Figure S1).

**Figure 4:**
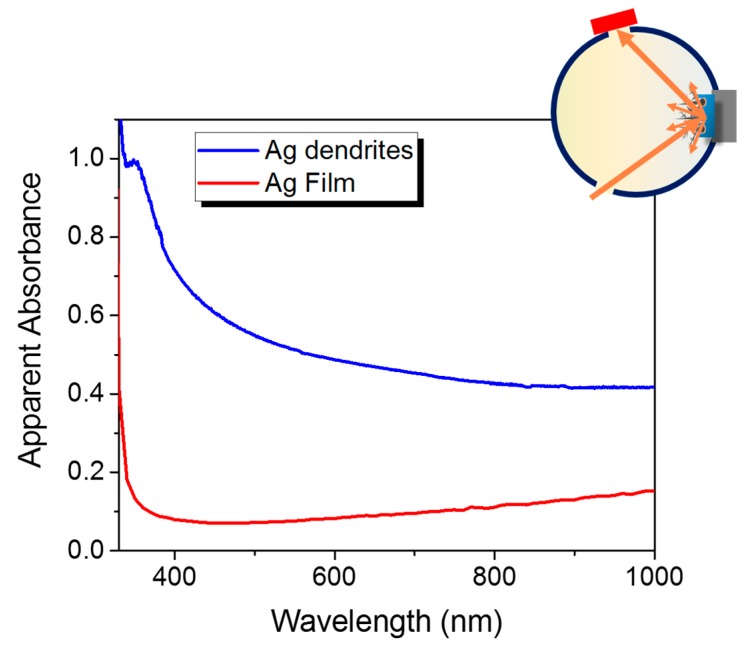
Ag plasmon resonance. Apparent absorbance spectra of the Ag reference film [55,56] (red spectrum) and Ag fractal dendrites (blue spectrum) obtained from the reflectance spectra carried out with an integrating sphere.

Figure 4 shows the plasmon properties of the Ag fractal dendrite material (in blue) compared to the optical properties of the Ag bulk continuous layer taken from reference [55,56] (in red). In particular, their apparent absorbances were obtained as log(100/R%) from the reflectance R% measurements, and the values representing the extinction signals of the samples are plotted (see Material and Methods for experimental details). In the case of Ag dendrites, an overall broad resonance, which extended to all the visible range up to the near infrared region, was observed. This broadness is typical of fractal aggregates, which, in the case of dendrites, are composed of branches with different lengths and widths that have varying gap sizes. Each length scale leads to a slightly different resonance position [27] thus, whatever the excitation wavelength in this range, it will always be possible to match a plasmon resonance condition. In particular, for the SERS experiments, we used the 633 of He–Ne laser as excitation wavelength because it is included in the broad plasmon resonance and, at the same time, allowed us to correctly measure the enhancement factor. Indeed, the evaluation of the EF implied a detectable Raman spectrum of the lysozyme in liquid as reference, and the 633 nm wavelength permitted us to observe the lysozyme Raman fingerprint in liquid, thus minimizing the fluorescence background that appeared as an easily removable plateau.

### 3.3. SERS Performances

**Figure 5:**
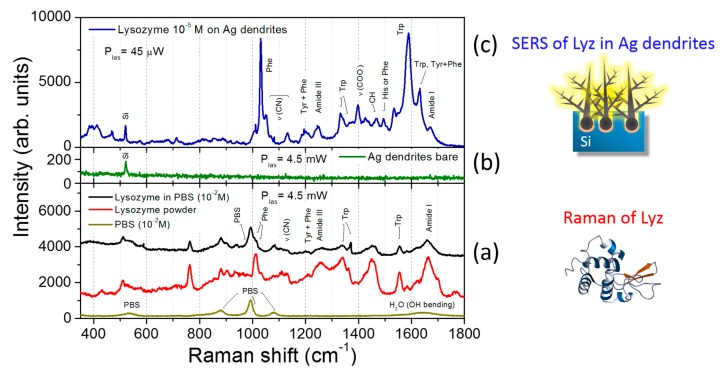
Surface-enhanced Raman scattering (SERS) performance of Ag dendrites. (**a**) Comparison between the Raman spectra of the lysozyme dry powder (red line), the pure phosphate buffer saline (PBS) solution (dark yellow line), and lysozyme in the PBS solution at 10 mM (black line). Notice that for these latter two spectra performed onto liquid samples, we used glass microcells as holders. (**b**) shows the Raman spectrum acquired on the bare Ag dendrites, while the blue spectrum in (**c**) shows the SERS response of 10^−5^ M of lysozyme onto the Ag dendrite platform. For all the spectra, we used the same 100× microscope objective with an integration time of 30 s. We used a laser power of 4.5 mW for the Raman spectra in (**a**,**b**) and 45 μW in the spectrum shown in (**c**).

**Figure 6:**
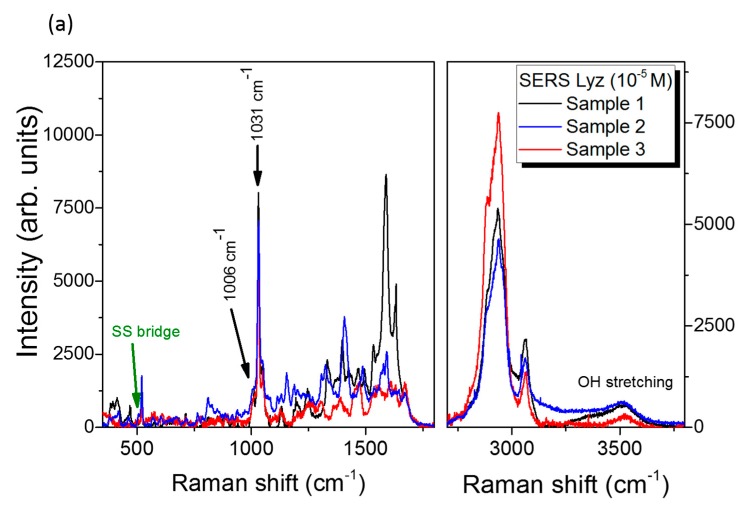

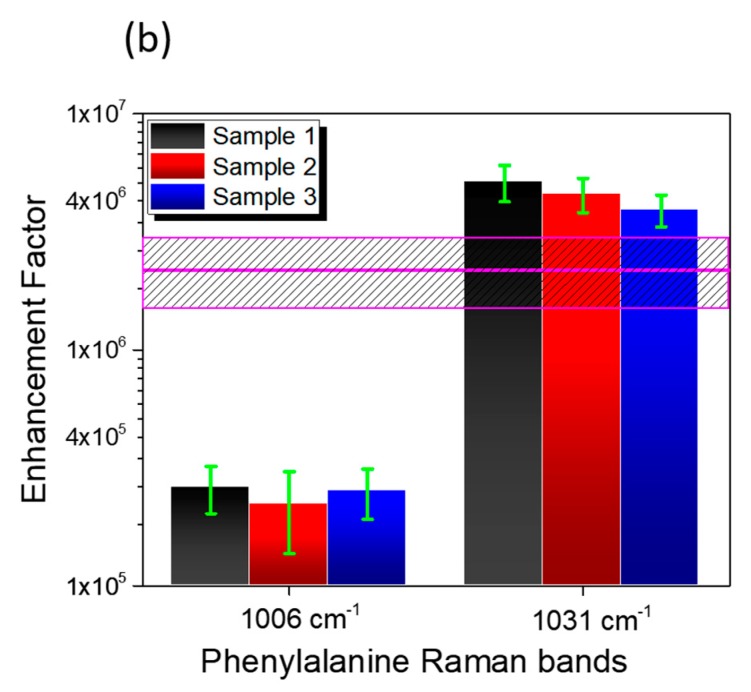
SERS signal from lysozyme target protein (Lyz) hydration region. (**a**) SERS spectra of lysozyme (10^−5^ M) measured on three different samples, as representative for the ensemble of measurements, presented in the low and high frequency regions. (**b**) Enhancement factors measured on the three spectra reported in (**a**) for the phenylalanine (Phe) peaks at 1006 and 1031 cm^−1^. The magenta line and dashed rectangle indicate the enhancement factors (EFs) calculated average value. For all the SERS spectra in (**a**), we used the same 100× microscope objective with an integration time of 30 s and a laser power of 45 μW.

The SERS performances of Ag fractal dendrites were investigated by using the lysozyme (Lyz) target protein, as reported in Figure 5. Different solutions at variable concentration of lysozyme in PBS were prepared, and Raman and SERS measurements were carried out as described in the Materials and Methods section. In Figure 5a, the Raman spectra of Lyz—dissolved in water (black line) and in powder (red line)— and of pure liquid PBS (dark yellow line) are shown. In particular, we note that the spectrum of Lyz dissolved in PBS was clearly detectable starting from a concentration of 10 mM. Here, the protein fingerprints, recognizable from the comparison with the Raman spectrum of Lyz powder placed on a microscope glass, emerged from those typical of the buffer solution (at 880, 993 and 1080 cm^−1^). The Raman signal from the bare fractal Ag dendrites without protein is also reported in Figure 5b (green spectrum), where only a peak at 520 cm^−1^ can be observed on a flat background due to the underlying Si substrate. In Figure 5c, the SERS spectrum of Lyz in PBS at 10^−5^ M deposited onto the silver dendrite platform is shown (blue line). We noticed that the Lyz contributions overcame the spectral signatures coming from the buffer, despite the experimental conditions adopted for SERS experiments for which 1) the laser intensity was set at two orders of magnitude lower than in the Raman experiment case, and 2) the molar concentration was three orders of magnitude lower, as specified in the figure caption. The Lyz spectral fingerprint consisted of many intense vibrational modes; among them, the bands of the amide III (1240–1270 cm^−1^), the COO^−^ stretching at 1398 cm^−1^ III, and the amide I at 1665 cm^−1^ are well visible. On the other hand, in the region around 1000 cm^−1,^ we could discern phenylalanine (Phe) and tryptophan (Trp) ring breathing bands from the PBS signal; their characteristic signatures in the spectral region between 1550 and 1620 cm^−1^ were also enhanced [57]. The aromatic side chain groups were indeed more subjected to SERS amplification, as has been extensively reported in many experiments in the literature [50,57]. This occurrence is probably due to the electrostatic affinity of the aromatic rings with the metal surface. Indeed, a delocalized charge distribution around the ring itself allows for a marked electronic mobility, which favors the electrostatic interaction with the metal substrate. This implies that these molecular groups, laying in close proximity to the nanostructured metal surface, benefit from greater field amplification. On the other hand, the aromatic residues are exposed in the outer part of the protein, and this occurrence could indicate its unfolding. However, in the SERS spectra, we could appreciate the S–S bond peak at 507 cm^−1^ and the amide bands in the spectral region between 1640 and 1680 cm^−1^, indicating a folded chain [58]. In Figure 6, three different samples, representative of the ensemble of measurements, are shown in the low and high frequency spectral regions (Figure 6a). In the latter, the presence of water shows the hydrated condition of Lyz, thus suggesting a scenario for which the protein was partially distorted in proximity of the metal surface due to the hydrophobic interaction with the aromatic residue side chains while maintaining the hydration condition around it. This thesis could also be confirmed by the slight differences appearing between the three SERS spectra shown in Figure 6a due to the soft blinking mechanism of the proteins close to the metal surface.

An estimation of the SERS enhancement factor (EF) gives information about the strong SERS performances of the silver dendrite platform. In order to estimate a representative EF for our SERS Ag dendrite platform, we analyzed the Phe ring breathing bands for the three samples shown in Figure 6a. These bands, marked with black arrows in the figure, were due to the symmetric ring breathing mode at 1006 cm^−1^ and the in-plane ring CH bending at 1031 cm^−1^ [50,59,60,61]. For each sample, we calculated the EF of the two Phe ring bands by comparing the integrated intensity for SERS and Raman spectra, as shown in Figure S2 of the Supplementary Information, where the Sample 1 fitting procedure is reported. The EF evaluation accounted for the scaling of the molecular concentration in the solutions, for the laser power density in the objective focus, and for the integration time, as reported below. Upon averaging the three spectra, we obtained an enhancement of ~2.5 × 10^5^ and ~4.5 × 10^6^ for the Phe bands at 1006 and 1031 cm^−1^, respectively, from which we deduced an averaged EF for the Ag dendrite SERS platform of ~2.4 × 10^6^ (see Figure 6b and Table 1). We can hypothesize that the difference of EFs in the Phe modes was due to the particular orientations or conformations of the Phe ring on the Ag surface, which inhibited the Phe symmetric ring breathing mode [60] with respect to other ring modes. In a SERS effect, the locally enhanced electric field is strongly polarized along the nanocavities axes [9,62,63]. In such a case, due to the so-called SERS surface selection rules for the SERS electromagnetic enhancement [64], the molecules that have polarizability tensors in the direction of nanocavities axes experience the highest change in the electron distribution. As a consequence, the Raman vibrational modes of Phe along the cavities axes are more enhanced; otherwise, they have low intensities when perpendicular [59,65]. In our particular case, the nanocavities were randomly oriented in the space, and we can imagine that the aromatic rings disposed inside the nanocavities (hot spot regions) mainly tilted with respect to the nanocavities axes (which means that they were flat to the metal surface).

The expression used for the assessment of the enhancement factor,

$$EF=\left(I_{SERS}/N_{SERS}\right)\times \left(N_{Raman}/I_{Raman}\right),$$

implies the knowledge of *N_SERS_* and *N_Raman_*, which are the number of probed molecules in the scattering volume for SERS and Raman experiments, respectively, calculated from the following expressions:

$$N_{SERS}=N_{Avo}\times c_{SERS}\times V_{las}$$

$$N_{Raman}=N_{Avo}\times c_{Raman}\times V_{las}$$

where *N_Avo_* is the Avogadro number, *c_SERS_* and *c_Raman_* are the molar concentration in the SERS and Raman case, respectively, and *V_las_* is the volume probed by the microscope objective that we assumed to be the same in both experiments. This approach revealed conservative calculations for the enhancement factor, since in the SERS experiments, the probed volume was occupied, to a large extent, by the silver dendrites, thus leading to an evaluated EF probably lower than the real one; this was calculated at the end as follows:

$$EF=\left(I_{SERS}/c_{SERS}\right)\times \left(c_{Raman}/I_{Raman}\right)$$

**Figure 7:**
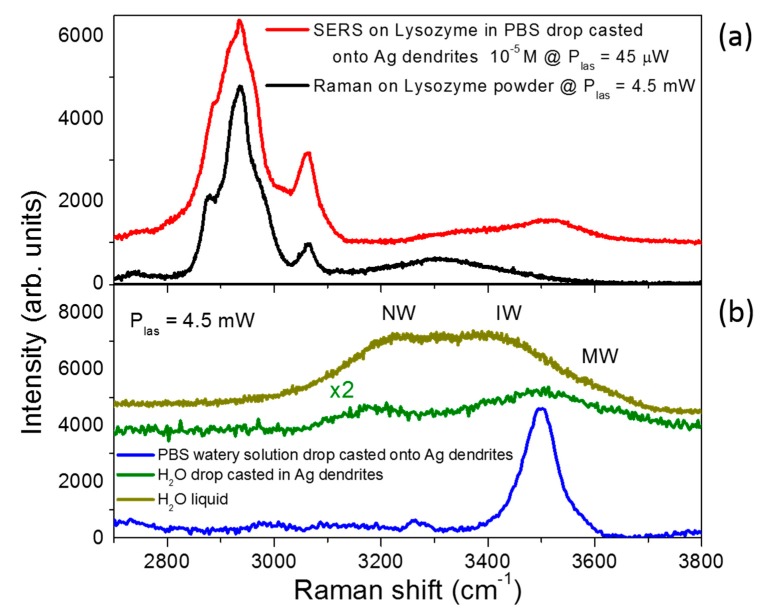
SERS signal from Lyz in the hydration region. Highlight of the SERS fingerprint of lysozyme onto Ag dendrites (in red) versus the lysozyme powder Raman spectrum (in black) in the water hydration region. For a better comparison, the spectra of the PBS solution drop casted onto the Ag dendrites (in blue), the water drop casted onto the Ag dendrites (in green), and liquid water are also presented (dark yellow). For all the spectra, we used the same 100× microscope objective with an integration time of 30 s, while we adopted a laser power of 4.5 mW for the Raman spectra and a laser power of 45 μW for the SERS one (red line in (**a**)). For the spectrum measured of the liquid sample (dark yellow line in (**b**)), we used glass microcells as sample holders.

In Figure 7, we show a qualitative comparison between the SERS spectrum of lysozyme in PBS at 10^−5^ M and the Raman spectrum of protein powder in the region between 2700 and 3800 cm^−1^. Here, C–H, N–H, and O–H vibrations bands are usually detected; the C–H and N–H atom groups generally belong to the protein, while the O–H groups are attributed to water molecules. In the SERS spectrum, we noticed a rearrangement in the hydrogen bond network. Indeed, we distinguished the presence of O–H stretching modes in the range between 3400 and 3700 cm^−1^, which arose from a distorted tetrahedral H-bonded network and the free O–H groups belonging to water shells [66,67] that were not detectable in the Raman spectrum of the dried protein.

To better understand the molecular arrangement in this spectral range, we further show the spectrum of the PBS solution and that of liquid water —both drop-casted onto dendrite samples— in Figure 7.

All these spectra show the different shapes of the OH stretching contributions in the various cases, and the coming out scenario confirms that the water content present in Lyz SERS spectrum was mainly due to a distorted H-bonded network of water molecules surrounding the protein, then representing its hydration shell. As highlighted in literature, the dynamics of water in the hydration shell of the proteins is slower than in the bulk [67,68] because the water molecules will not be strictly arranged in a tetrahedral organization. In the O–H stretching region, this hydration shell gives rise mainly to the so-called intermediate water (IW) band at about 3450 cm^−1^ due to water molecules with a low degree of connectivity and a coordination number close to three [69], and to the less intense loosely bonded water band, or multimer water (MW), at about 3600 cm^−1^. Details on the dominating O–H stretching features of pure liquid water, identified in terms of the main degrees of connectivity between molecules, are shown in Figure S3 of the Supplementary Information. On the other hand, the pure liquid water drop casted onto the silver dendrites (green line in Figure 7b) showed the typical Raman spectrum of confined water [69], confirming the thesis of the presence of a distorted O–H network confined to the pores of the plasmonic sample. By comparing this spectrum with that of the pure liquid water (dark yellow), we noticed that a minor part of water molecules was involved in tetrahedral H-bonded network that gave rise to the so-called network water (NW) band centered at about 3250 cm^−1^ (high degree of connectivity) [70]. As expected, the spectrum of the PBS watery solution drop casted in silver dendrites (blue line) showed a band arising from the convolution of the IW band together with the MW band.

The results clearly indicate that, despite the fact that the droplet containing the protein was left to dry after its deposition onto the SERS substrate, lysozyme laid in a hydration condition. The material porosity played a key role in this scenario; indeed, the hypothesis was that lysozyme remained in its natural hydration habitat due to the presence of confined regions inside the fractal silver dendrite material, which was subjected to plasmonic enhancement effects.

This latter occurrence offers the possibility to access protein activity by means of Raman spectroscopy that takes advantage of the signal enhancement, this being a desired opportunity in biomedical field. Our 3D Ag dendrite SERS platform lacks selectivity at the present state. Nonetheless, fractal silver dendrites could be used as SERS sensors for an assay of the specific proteins contained in bioliquids after the suitable functionalization of the metal surface with a tailored bioreceptor. In this context, the use of aptamers could commute the SERS platforms in a real sensor with a high affinity to the targeted protein, gaining selectivity and maintaining sensitivity [17].

## 4. Conclusions

In summary, we have presented a simple chemical deposition method for the fabrication of a Ag dendrite fractal network for the SERS detection of biomolecules. The Ag dendrite fractal nanostructures exhibited a large enhancement of the Raman signatures up to 2.4 × 10^6^. This hierarchical structure, characterized by nanocavities of various dimensions where a watery solution is likely confined, is a promising platform to study the biomolecules activity in their natural environment.

## Figures and Tables

**Table 1:** All the measured EFs calculated from the spectra in Figure 6 (**a**) and reported in Figure 6 (**b**) are resumed in Table 1 for the Raman peaks at 1006 and 1031 cm^−1^.

Enhancement Factor	Peak 1006 cm^−1^	Peak 1031 cm^−1^
Sample 1	2.6 ± 0.6 × 10^5^	5.1 ± 0.9 × 10^6^
Sample 2	2.2 ± 0.8 × 10^5^	4.6 ± 0.8 × 10^6^
Sample 3	2.5 ± 0.6 × 10^5^	3.9 ± 0.6 × 10^6^
Average	2.4 ± 0.8 × 10^6^	

## Supplementary Materials

The following are available online at https://www.mdpi.com/2079-4991/9/11/1630/s1, Figure S1: Contaminations study by Raman investigation of Ag dentrites; Figure S2: SERS and Raman detection of Lyz in the aromatic residues ring breathing modes spectral region; Figure S3: Raman spectrum of liquid water.
