# Supplementary material for: Fractal Silver Dendrites as 3D SERS Platform for Highly Sensitive Detection of Biomolecules in Hydration Conditions
Source: Nanomaterials (Basel). 2019 Nov 16;9(11):1630. doi: 10.3390/nano9111630 (PMC6915472; doi:10.3390/nano9111630)
Supplement: Supplementary file 1 [file nanomaterials-09-01630-s001.pdf]

Supplementary Materials: Fractal Silver Dendrites as 3D SERS Platform for Highly Sensitive Detection of Biomolecules in Hydration Conditions

Maria José Lo Faro [1,2,3], Cristiano D'Andrea [4], Antonio Alessio Leonardi [1,2,3], Dario Morganti [1,2], Alessia Irrera [2,*] and Barbara Fazio [2,*]

[1] Dipartimento di Fisica e Astronomia, Università di Catania, via S. Sofia 64, 95123 Catania, Italy; mariajose.lofaro@dfa.unict.it (M.J.L.F.); antonio.leonardi@ct.infn.it (A.A.L.); dario.morganti@ct.infn.it (D.M.)
[2] CNR - IPCF, Istituto per I Processi Chimico-Fisici, viale F. Stagno d'Alcontres 37, 98158 Messina, Italy
[3] CNR - MATIS IMM, Istituto per la Microelettronica e Microsistemi, via S. Sofia 64, 95123 Catania, Italy
[4] CNR - IFAC, Istituto di Fisica Applicata "Nello Carrara", Via Madonna del Piano, 10, I-50019 Sesto Fiorentino, Italy; c.dandrea@ifac.cnr.it
* Correspondence: irrera@ipcf.cnr.it (A.I.); fazio@ipcf.cnr.it (B.F.); Tel.: +39-090-3976-2266 (A.I.); +39-090-3976-2246 (B.F.)

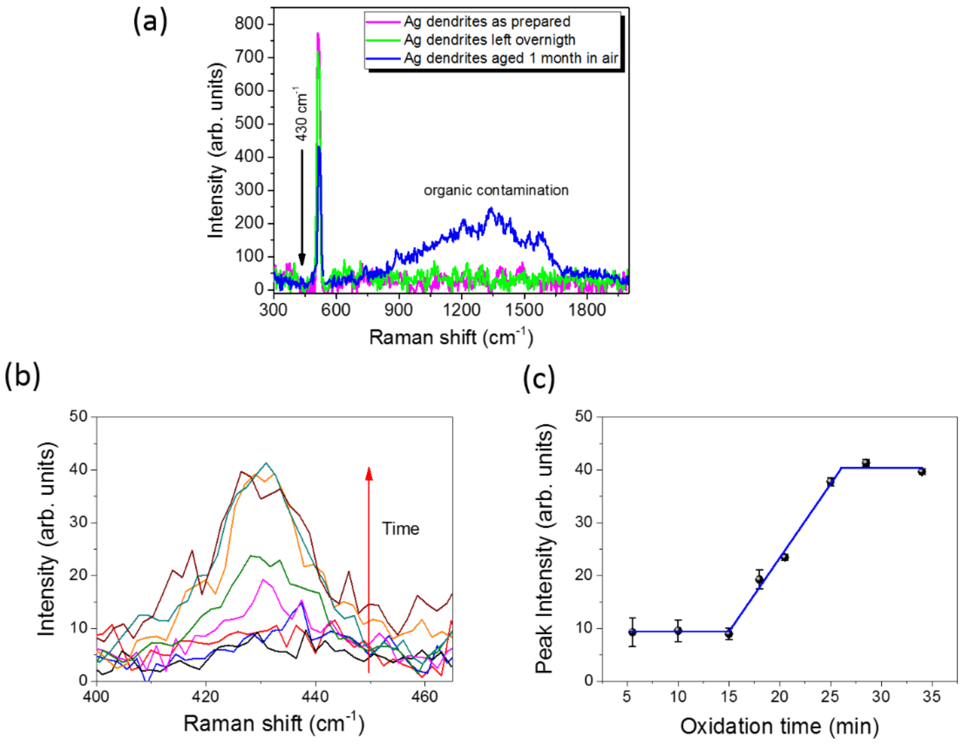

Figure S1. Contaminations study by Raman investigation of Ag dentrites**.** (**a**) Raman spectra of Ag dendrites as prepared (magenta spectrum), Ag sample left overnight under the chemical hood (green spectrum) and exposed to air for 1 month (blue one). (**b**) Raman spectra of Ag dendrites oxidation as a function of time induced by UV cleaner and (**b**) AgO peak intensity evolution from 5 min to 35 min.

In the case of silver SERS platforms, a major drawback is related to the evolution of their morphological and optical properties due to ambient exposure leading to oxidation and organic contamination. Both phenomena detrimentally affect the SERS performances of the system. In particular, the oxidation layer affects the size, generally inducing a plasmon resonance shift and diminishing the effective coupling between the localized plasmon resonance and the attached molecule.

We investigated the presence of organic contamination by Raman spectroscopy performed onto as prepared Ag dendrites (magenta spectrum), Ag dendrites left overnight under a chemical hood (green spectrum), and samples aged in air for 1 month (blue one) as presented in the supplementary Figure S1 (a). The comparison of the Raman spectra shows that no organic contamination is attested onto as prepared Ag dendrites, possibly due to the presence of the hydrofluoric acid during the growth. Similarly, no contaminants are observed in Ag dendrites exposed overnight in a chemical hood. On the contrary, samples stored in air for one month clearly attest the presence of the typical carbon related contamination band in the region from 1000 to 1600 cm^{-1}.

In our case, after the synthesis of Ag dendrites we do not observe the typical oxidation peak arising from the silver oxide presence attested at 430 cm^{-1}. In particular, we deal with a nanostructured material made of branches with a broad distribution in size. This leads to a broad plasmon resonance for which the eventual mechanism of oxidation does not critically affect the optical response. Nonetheless, to prevent their chemical modification, we preserved the samples in a chamber under dry and low vacuum conditions (stored samples). As well known, UV-cleaning procedure in ozone rich environment can efficiently remove the carbon contaminations. On the other hand, this induced oxidation modifies the surface chemistry leading to the formation of silver oxide. Therefore, we monitored the evolution of the oxide layer as a function of UV-oxidation exposure from about 5 to 35 min through Raman spectroscopy. In particular, we study this phenomenon from the intensity of the typical AgO Raman peak (at 430 cm^{-1}), as reported in the supplementary Figure S2, where both the (a) silver oxide Raman spectra and (b) its intensity trend are shown overtime. As visible, no silver oxide peak is attested for UV oxidation time below 15 minutes, above this threshold an oxide layer is formed, as confirmed by the Raman peak intensity increment. After 25 minutes of UV cleaning, the AgO intensity signal saturates, indicating the complete oxidation of Ag dendrites by using a UVO-Cleaner (Jelight 42-220, Irvine, CA, USA) at a 28 mW/cm2 at the wavelength of 254 nm.

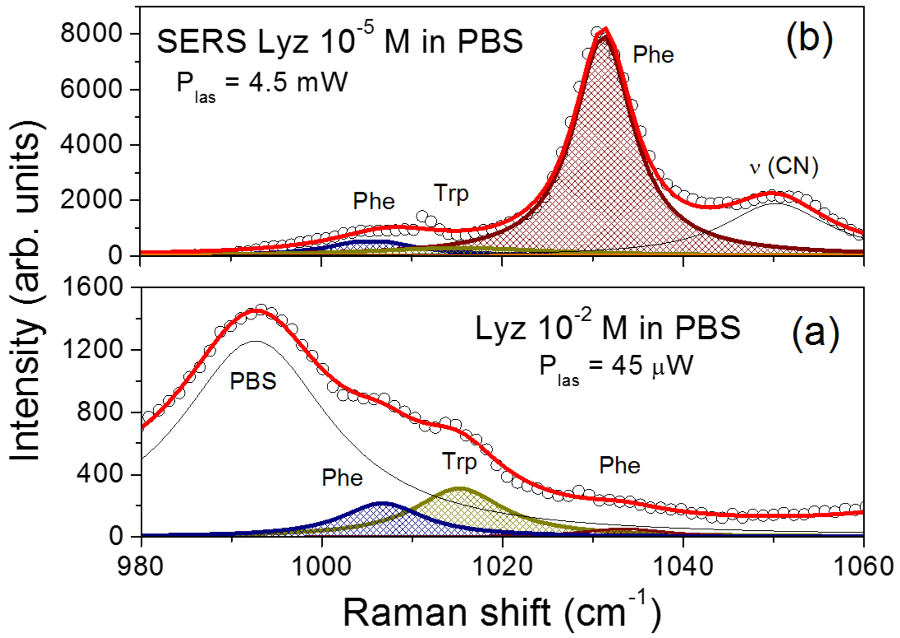

Figure S2. SERS and Raman detection of Lyz in the aromatic residues ring breathing modes spectral region. In the zoom in the region around 1000 cm^{-1} (**a**) Raman spectrum of liquid Lyz 10 mM in PBS and (**b**) SERS spectrum of Lyz 10^{-5} M in PBS deposited into silver dendrites are shown (hollow symbols). The spectra highlight the Phe (1006 and 1031 cm^{-1}, blue and red coloured area respectively) and Trp (1014 cm^{-1}, dark yellow coloured area) ring breathing peaks, for which Lorentzian fitting have been adopted to separate them. For the two spectra we used the same 100× microscope objective, integration time of 30s, while we adopted a laser power of 4.5 mW for the Raman spectrum (**a**) and

45 μW for the SERS one (**b**). For the liquid Lyz 10 mM in PBS sample we used a glass microcell as holder.

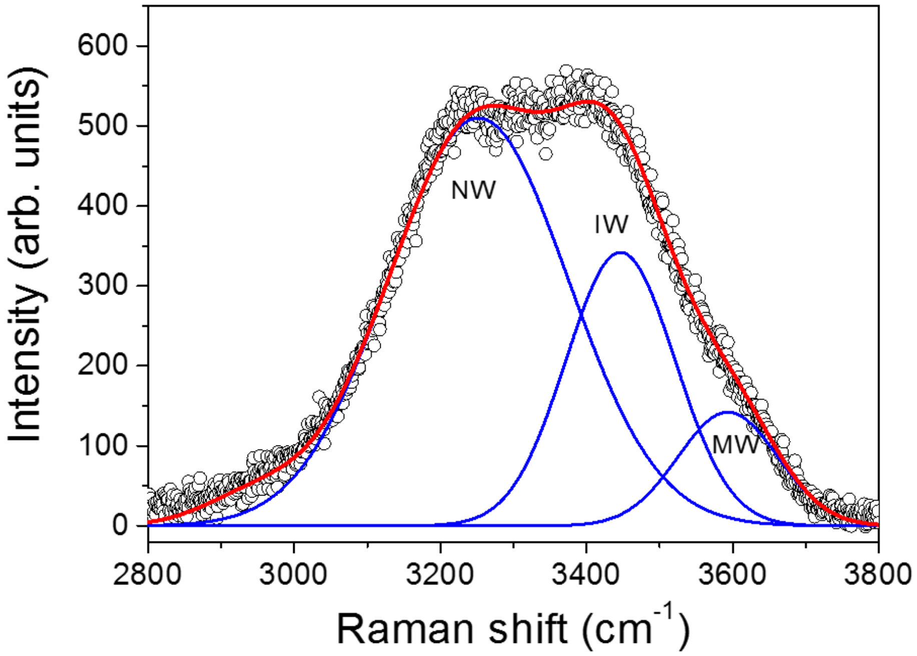

Figure S3. Raman spectrum of liquid water. Raman spectrum of liquid water (circle) and its respective fit curve (red line). In blue the fit curves obtained by taking into account three O–H stretching Gaussian distributions are shown. These Gaussian shapes identify the main degrees of connectivity established between the water molecules: NW, IW and MW. We acquired the spectrum at an integration time of 30 s, by using a laser power of 4.5 mW focused by means of a 100× microscope objective into the sample. The liquid sample was put into a glass microcell.
